# Provision of externally accredited immunisation certification within an Australian Professional Pharmacy University programme: a survey of graduates on benefits and outcomes

**DOI:** 10.1093/ijpp/riac016

**Published:** 2022-03-09

**Authors:** Nijole Bernaitis, Gary Grant, Susan Hall

**Affiliations:** School of Pharmacy and Medical Sciences, Griffith University, Gold Coast Campus, Gold Coast, QLD, Australia; School of Pharmacy and Medical Sciences, Griffith University, Gold Coast Campus, Gold Coast, QLD, Australia; School of Pharmacy and Medical Sciences, Griffith University, Gold Coast Campus, Gold Coast, QLD, Australia

**Keywords:** professional training, education, health promotion, professional role

## Abstract

**Objectives:**

In Australia, pharmacists may become authorised immunisers by obtaining additional credentialling from certified providers. Some Australian Universities are providing externally accredited immunisation training to final year pharmacy students. Student satisfaction has been demonstrated, but graduate views on benefits and outcomes are yet to be determined. The aim of this study was to evaluate graduates’ perceptions of providing an accredited immunisation certification during their University pharmacy programme.

**Methods:**

A survey was sent to Griffith University pharmacy graduates of 2016–2020 inclusive. Respondents who completed the accredited immunisation training at University were asked to rank their agreement with five statements on a five-point Likert scale (1 = Strongly Disagree, 5 = Strongly Agree). Further information, including free-text responses, was collected on current area of practice and involvement in vaccinations.

**Key findings:**

Of the 46 graduates who completed the survey, 42 (91.3%) completed the accredited immunisation training at University. Statements that the accredited immunisation training was considered a valuable additional offering to the pharmacy programme and the time commitment was worthwhile resulted in a mean agreement of 4.74 ± 0.73 and 4.64 ± 0.76, respectively. The majority of respondents (*n* = 27, 58.7%) were providing immunisations on a daily, weekly or monthly basis with over half reporting becoming more actively involved in immunisation due to COVID-19.

**Conclusions:**

Pharmacy graduates valued completing an externally accredited immunisation training within their University programme and reported benefits to their employability and current roles. Incorporating externally accredited training into the curriculum can ensure graduates are prepared and skilled in continually expanding roles for pharmacists.

## Introduction

Immunisation is integral to communicable disease control worldwide by reducing the incidence of disease.^[1]^ In Australia, pharmacists have always been actively involved in immunisation through roles as vaccine educators, advocates and distributors.^[2]^ In 2014, in line with international practice, Australian pharmacist roles expanded to include the provision of pharmacist-administered immunisation services.^[3]^ Provision of immunisations through Australian pharmacies provided improved convenience and accessibility to immunisation services with the overall benefit of increased vaccination rates.^[4]^ During the COVID-19 pandemic, in addition to the crucial role in supporting their patients, community pharmacies began participating in the administration of the COVID-19 vaccine.^[5]^ In September 2021, community pharmacies across Australia administered 400 000 COVID-19 vaccines demonstrating capacity and capability to effectively deliver important vaccination services.^[6]^ As of 6 December 2021, Australian pharmacy primary care providers had delivered more than 2.4 million vaccine doses.^[7]^

Most community pharmacies provide vaccinations as part of their service model and employ authorised immunisers for this role.^[8, 9]^ Australian registered pharmacists can be authorised immunisers by obtaining additional credentialling from certified providers.^[10]^ Components of the credentialling include prerequisite online modules with associated assessment together with a practical workshop and assessment focussing on injection techniques. Successful completion of this training course combined with evidence of current first aid, cardiopulmonary resuscitation and anaphylaxis certificate enables the issue of a Certificate of Attainment which qualifies an Australian pharmacist to administer vaccines in accordance with legislative guidelines. In some Australian states, such as Queensland, provisionally registered (or trainee/intern) pharmacists who have the immunisation Certificate of Attainment may administer vaccines under the direct and personal supervision of a pharmacist.^[11]^

Graduating pharmacy students can be well prepared for administering immunisations by incorporating immunisation education in the curriculum and developing appropriate practical skills.^[12, 13]^ Since 2016, Griffith University (GU) School of Pharmacy and Medical Sciences has been providing final year pharmacy students the option of completing an externally accredited immunisation training. Successful completion of the accredited training, that is, online modules and practical workshops from the certified provider, enables graduates to obtain the immunisation Certificate of Attainment immediately upon provisional registration. This potentially benefits the graduate in terms of employability and development of skills but also the health profession by ensuring graduating pharmacists are practice-ready and the immunisation workforce capacity is expanded.

Over the past 5 years, around 70% of final year GU pharmacy students have undertaken the optional externally accredited immunisation training so they could obtain the official immunisation Certificate of Attainment upon provisional registration. The GU School of Pharmacy & Medical Sciences has been providing this optional certified training in addition to the pharmacy curriculum to enhance graduate skills, employability and scope of practice. Australian studies have demonstrated student satisfaction with provided immunisation training together with increased skills and confidence with injection techniques.^[14–16]^ However, to our knowledge, there are no studies evaluating graduate perceptions of the benefits of completing this certified training in addition to the pharmacy curriculum. Therefore, the aim of the research was to evaluate graduates’ perceptions of completing the additional externally accredited immunisation certification during their University pharmacy programme. Secondary objectives were to evaluate the potential impact on their current roles in pharmacy practice, including during the current pandemic environment.

## Methods

### Study design

This was a cross-sectional survey of pharmacy graduates from one Australian University in the years 2016–2020 inclusive, that is, graduates who had been provided the option to complete the externally accredited immunisation training while at University. GU offers both a Bachelor of Pharmacy (B.Pharm) and Master of Pharmacy (M.Pharm) programme and graduates from both programmes were included in the research.

### Data collection methods

A survey for self-completion was developed to be distributed online using Microsoft Forms. The questions aimed to collect information regarding the current workplace of the pharmacy graduates and their perceived benefits and outcomes from the provided immunisation training. The first five questions collected demographic information regarding the graduate including their University programme, year of completion and their current area of practice as a pharmacist. Question 6 asked if students completed the externally accredited immunisation training at University with the survey branched at this point according to the response. Respondents who did not complete the accredited immunisation training at University were asked for reasons why they chose not to undertake this training but also if they had now completed an accredited immunisation training. Respondents who had completed the accredited immunisation training were asked five questions regarding the provided training with responses rated on a five-point Likert scale, with 1 being Strongly Disagree and 5 being Strongly Agree. All respondents who had completed the accredited training (at University or post-University) were asked about the frequency of providing immunisations in their current role which could be rated as daily, weekly, monthly or less than monthly. The remaining questions provided for open-ended free-text responses about the participant’s current role in providing COVID-19 immunisations including ‘Have you become more actively involved in immunisations due to COVID-19?’, ‘Do you currently have a role involving COVID-19 immunisations?’, ‘Do you intend to become involved in COVID-19 immunisations?’ The final question provided an invitation for any comments in relation to externally accredited immunisation training. The survey form (Supplementary data) asked respondents to read each question carefully and answer as openly as possible, but there was the potential for the form to be submitted without all questions answered. The research team tested the survey to ensure correct branching and completion of relevant questions for all response options.

### Ethics approval

Ethics approval was obtained from the GU Human Research Ethics Committee: GU Ref No. 2021/537.

### Survey dissemination

The Executive Officer of the GU School of Pharmacy and Medical Sciences sent an e-mail to B.Pharm and M.Pharm graduates of 2016–2020 inclusive, that is, graduates who had been provided the option to complete the externally accredited immunisation training. The e-mail outlined the research and provided a one-user link to the questionnaire on Microsoft Forms with completion of the questionnaire taken as indicating informed consent. The e-mail was sent to all potential participants a total of three times, namely 2 August, 17 August and 4 September 2021 with the survey closed on 25 September 2021. Responses provided were anonymous with no identifying information collected.

### Data analysis

Researchers accessed anonymous responses through Microsoft Forms with responses downloaded to Microsoft Excel. Both complete and partially completed forms were eligible for analysis. Questions with responses rated on a five-point Likert scale were coded with 1 being Strongly Disagree and 5 being Strongly Agree and then downloaded into SPSS Statistics 26 for analysis. Collated responses were represented as number (percentage) for categorical data and mean (standard deviation) for Likert scale responses as calculated by SPSS Statistics 26. Qualitative data were thematically analysed, and direct responses were utilised as appropriate.

## Results

### Respondent characteristics

A total of 270 graduates were contacted via e-mail, with eight (2.9%) e-mails undeliverable. Of the 262 graduates contacted, 46 (17.5%) completed the survey. All questions had been completed by all those who responded. The majority of respondents were currently working as pharmacists (*n* = 43, 93.5%) in community pharmacy (*n* = 33, 71.7%) and metropolitan locations (*n* = 26, 56.5%) (Table 1).

**Table 1:** Information from survey responses on pharmacy graduates and current workplace (*N* = 46)

Variable	Number
Year of graduation	
2020	9
2019	6
2018	9
2017	11
2016	11
Programme	
Bachelor of Pharmacy	27
Master of Pharmacy	19
Current employment	
Pharmacist	43
Intern pharmacist	2
Other	1
Area of practice	
Community	33
Hospital	11
Research	1
Other	1
Location of practice	
Metropolitan	26
Rural	11
Other	9

### Main findings

**Figure 1:**
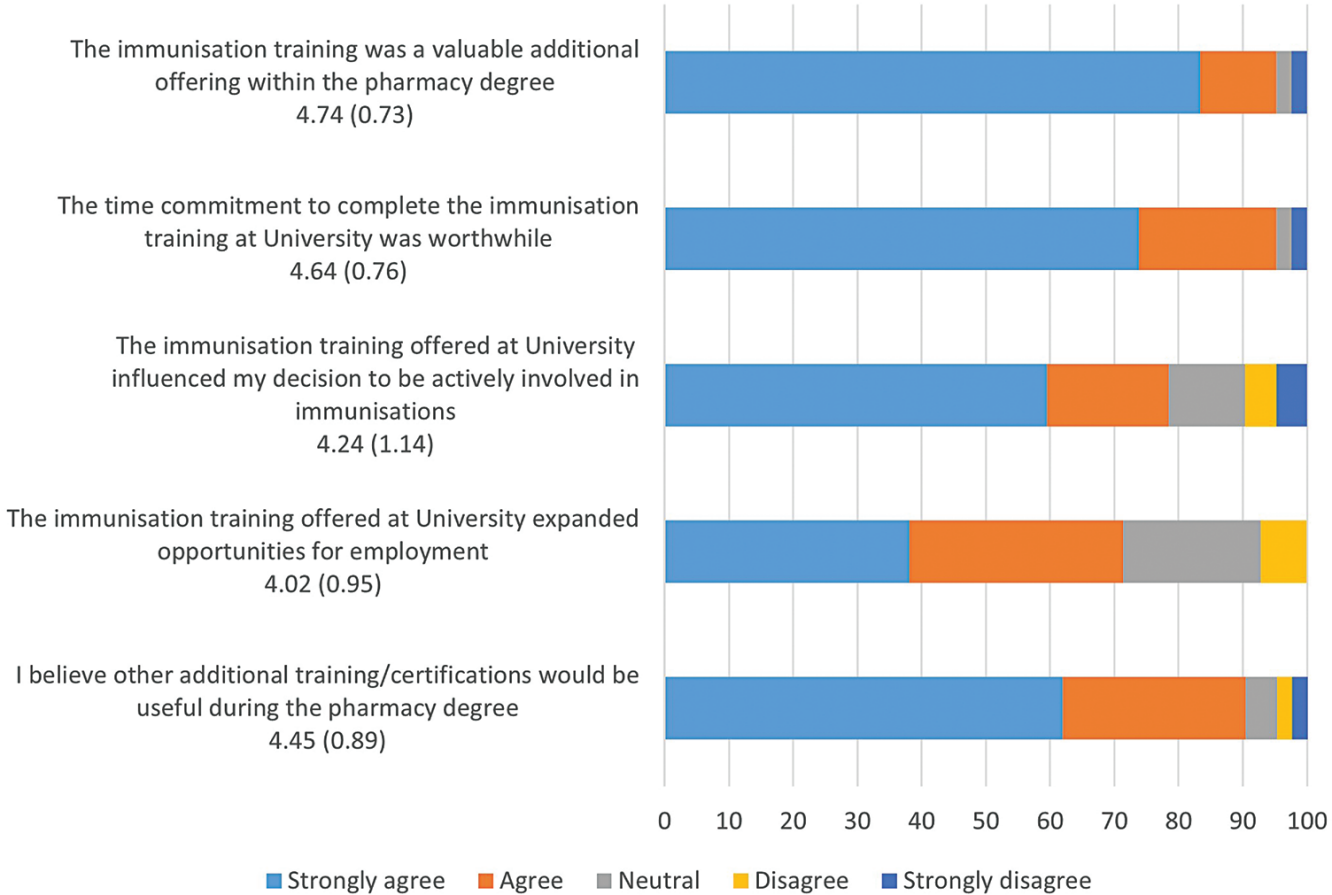
Responses (*N* = 42) to statements regarding immunisation training on five-point Likert scale with options of 1 = Strongly Disagree, 2 = Disagree, 3 = Neutral, 4 = Agree and 5 = Strongly Agree. Data represented as mean (standard deviation) after each statement.

A total of 42 (91.3%) respondents had completed the accredited immunisation training at University. Over 95% of respondents strongly agreed or agreed that the accredited immunisation training was a valuable additional offering to the pharmacy programme and the time commitment was worthwhile (Figure 1). Respectively, these two statements resulted in mean responses of 4.74 ± 0.73 and 4.64 ± 0.76 with responses to all five statements providing mean values above 4. Free-text comments (*n* = 18) were mainly positive regarding the timing, benefit and impact on employment with negative comments on administrative details of obtaining the certificate (Table 2).

**Table 2:** Free-text responses about the externally accredited immunisation training grouped according to the classification of response as positive or negative and theme with exemplar responses provided

Identified theme	Exemplar comments
Positive comments	
Benefit *N* = 11	‘It was a valuable asset going into the industry as an intern’ (P40) ‘I strongly believe that all students should be leaving university with a strong familiarity with all processes and procedures’ (P15) ‘I have discussed the fact we were given the opportunity to complete both vaccine training and mental health certificate with other pharmacy students and pharmacists and all have been very envious that was not offered in their own degrees’ (P21)
Employability *N* = 4	‘I found the vaccination training provided by the university helped me get a job faster’ (P9) ‘I found it immensely improved my employability in community pharmacy’ (P29) ‘Doing the training through my masters really set me aside from other interns at the time and helped me secure a pharmacist role’ (P8)
Timing *N* = 6	‘had I needed to wait for a course during my internship I would've missed most of flu season and been much less practised by the time I was registered’ (P29) ‘I utilised these valuable skills immediately during my internship and continue to do so every day’ (P16) ‘I was grateful to have had the opportunity to do this training at University and it was very worthwhile’ (P35)
Negative comments	
Timing *N* = 3	‘some community pharmacies will not allow interns to immunise due to insurance and staffing requirements’ (P28) ‘Now with the immunisation included in the intern training programs, unsure if a university based one would be of much benefit anymore’ (P8)
Administrative *N* = 2	‘was time consuming having to follow-up after grad to get the cert’ (P1) ‘I was unaware I had to pay a small admin fee’ (P26)

Three of the four respondents who did not complete the training at University had now completed the accredited immunisation training. Respondents reported providing immunisations daily (*n* = 17), weekly (*n* = 8) or monthly (*n* = 2). Respondents who reported providing immunisations on a less than monthly (*n* = 17) interval were predominantly hospital pharmacists (*n* = 10). Free-text comments regarding this included ‘As I work in hospital I am not able to give vaccines…When I was a community pharmacist I used it all the time’ (P40) and ‘There is a delegated covid pharmacist in my hospital who is vaccination trained, but it is not my role’ (P39).

The majority (*n* = 24) of respondents reported becoming more actively involved in immunisation due to COVID-19 with ‘vaccinations doubled in 2020 on any previous year in store’ (P10), ‘increasing uptake of whooping cough vaccine with annual influenza’ (P37) and ‘I have seen an increase in people being immunised in community pharmacy. Patients have commented on the ease of getting vaccinated in a community pharmacy setting’ (P20). Half of the survey respondents (*n* = 23) stated they had a role involving COVID-19 immunisation including ‘Implementation of policies and procedures, preparation of vaccinations and administration’ (P16), ‘Participating in the COVID-19 vaccine rollout has increased the number of immunisations I am providing on a daily basis. I have also been in charge of setting up and overseeing my pharmacy’s COVID-19 vaccination clinic’ (P16) and ‘providing 30+ covid immunisations daily and sometimes purely employed to vaccinate’ (P1).

## Discussion

This research found graduates believed an externally accredited immunisation certification in the final year of their University studies was a worthwhile, valuable addition to their pharmacy programme. Graduates reported the immunisaton training contributed to employability and current pharmacy role, with over half of the respondents now involved in COVID-19 immunisation.

This research was the first, to our knowledge, to survey graduates on their perceptions of including accredited immunisation training within a pharmacy programme. Limitations of the study are that the survey included graduates of only one Queensland University so may not be representative of other University graduates in Australia and internationally, especially due to the small response rate and sample size. Another limitation was that the vast majority of respondents had completed the immunisation training at University and thus students who did not undertake the University training were under-represented.

This research found the highest agreement with the statements that the immunisation training was a valuable additional offering and worthwhile time commitment. Consistent with this positive feedback, Mills *et al.* reported that incorporating immunisation training in the final year of pharmacy curriculum can improve student knowledge and self-perceived skills in vaccinations.^[15]^ Similar to this, Hope *et al.* found immunisation training increased injection skills and confidence, with particular benefit from the simulated experience with mannequins and placebo vaccines.^[16]^ The externally accredited immunisation training includes demonstration and assessment of practical skills, including reconstitution of vials and administration of both intramuscular and subcutaneous injections. This ensures the development of essential skills and competency in all practical techniques in preparedness for vaccination administration. Interestingly, all the Australian training content for the COVID-19 vaccination is delivered through an e-learning platform.^[17]^ One free-text comment from our survey stated ‘Would have loved more formal training in regards to covid vaccination. A government website course has its place but so too does practical practice’. This may reinforce the benefit of simulated training for the development of practical skills, particularly for pharmacist roles such as vaccinating.

This survey recorded agreement (i.e. mean above 4) with all five statements regarding the immunisation training, with the lowest mean score for the statement about expanded opportunities for employment. Despite this, the free-text comments were overwhelmingly positive regarding employment benefits and providing opportunities for increased roles. Negative comments appeared more focussed on administrative issues and the opportunity to provide immunisation while provisionally registered or intern pharmacists. Comments included ‘Now with the immunisation included in the intern training programs, unsure if a university based one would be of much benefit anymore’. However, completing the training in the final year of University offers the advantage of graduating with the additional qualification and being immediately ready to immunise which for most Australian graduates coincides with the influenza season. This potential benefit was reflected in a survey comment of ‘had I needed to wait for a course during my internship I would’ve missed most of the flu season and been much less practised by the time I was registered’. In contrast, another comment stated, ‘some community pharmacies will not allow interns to immunise…’. This raises concerns regarding the possible loss of competence and/or confidence in immunisation skills if not using them soon after acquisition and highlights the importance of appropriate timing of practical skills training. Bushell *et al.* noted varied approaches to timing when providing vaccination training within degree programmes but cited jurisdictional regulations and variations may influence choice, including the potential recognition of pharmacy students as vaccinators.^[14]^ Currently, GU provides this training as an optional offering in the final year of their programmes given that provisionally registered (or trainee/intern) pharmacists may administer vaccines under the direct and personal supervision of a pharmacist in Queensland.^[11]^ Similar to this, a further 11 Australian Universities provide vaccination training in the final year of their pharmacy programmes.^[18]^ Legislation in some Australian states and territories restricts the provision of accredited vaccination training to registered pharmacists only, that is, students and interns cannot undertake the training.^[19]^ While legislative and jurisdictional requirements may influence the ability of pharmacy students to obtain an official immunisation certification, it could be argued that all pharmacy students would gain essential knowledge and skills from immunisation training. In support of this, survey comments included ‘it’s essentially a prerequisite these days to be able to provide immunisations’. A 2009 American survey reported 10% of pharmacy students and 25% of faculty members believed an immunisation certificate programme should not be mandated in the pharmacy curriculum.^[20]^ However, given this was before the COVID-19 pandemic, it is possible that beliefs of staff and students have changed to better reflect the needs of the curriculum in current pharmacy practice. This study confirms the benefit of including immunisation training in the pharmacy curriculum. Further research is needed to determine the optimal level of immunisation training and the timing of any such training within pharmacy programmes.

Pharmacists have an important role in administering immunisations but the activities within community pharmacies and involvement in immunisation initiatives are not well documented.^[21]^ Our study found the majority of respondents who provided immunisations on a daily or weekly basis were community pharmacists, while hospital pharmacists reported providing immunisation less than monthly. Of note, half of the respondents stated they had a role involving COVID-19 immunisations while the majority became more actively involved in immunisations due to COVID-19. Free-text comments described increased uptake of numerous vaccinations which supports findings by Patel *et al.* whereby 96% of Australian pharmacists reported higher than expected demand for pharmacist-administered influenza vaccine due to the COVID-19 pandemic.^[22]^ COVID-19 has highlighted the possible consequences of infectious disease, the value of vaccines, and the inclination to receive vaccines from medical professionals whom patients trust.^[23]^ Pharmacists were well equipped to provide crucial services including vaccinations during this pandemic, but other expanded roles have emerged during the pandemic including telehealth services, virtual consultations and digital prescription handling.^[24]^ In the same way, the curriculum evolved to include immunisation training, pharmacy programmes require a dynamic response to include training and ensure the preparedness of pharmacy graduates in these ever-expanding roles.

## Conclusion

The COVID-19 pandemic increased the immunisation role for many pharmacists and highlighted the need to provide immunisation training to graduating pharmacy students. This novel survey of Australian graduates confirmed the benefit of incorporating immunisation training within the pharmacy curriculum in terms of employability and pharmacy practice roles, particularly during the COVID-19 pandemic. Further research involving early career graduates is encouraged to evaluate and improve the pharmacy curriculum and ensure graduates are prepared and skilled in constantly emerging roles for pharmacists.

## Supplementary Material

riac016_suppl_Supplementary_Material
