# Supplementary material for: Provision of externally accredited immunisation certification within an Australian Professional Pharmacy University programme: a survey of graduates on benefits and outcomes
Source: Int J Pharm Pract. 2022 Mar 9;30(3):273–8. doi: 10.1093/ijpp/riac016 (PMC9383585; doi:10.1093/ijpp/riac016)
Supplement: riac016_suppl_Supplementary_Material [file riac016_suppl_supplementary_material.docx]

**“Evaluation of the benefits and outcomes from externally accredited immunisation certification provided to final year pharmacy students”. (GU Ref No 2021/537)**

**Please read each question carefully and answer as openly as possible.**

Please indicate one response from the following questions:

1. Which year did you graduate from Griffith University?

2016 🞏

2017 🞏

2018 🞏

2019 🞏

2020 🞏

2. Which Griffith University program did you graduate from?

Bachelor of Pharmacy 🞏

Master of Pharmacy 🞏

3. Are you currently working as a pharmacist:

Yes 🞏

No 🞏

If no, when did you last work as a pharmacist? __________________

4. Which best describes your current area of practice:

Community 🞏

Hospital 🞏

Industry 🞏

Academia 🞏

Research 🞏

Other (please specify) _____________________________

5. Which best describes your current location of practice:

Metropolitan 🞏

Rural 🞏

Other (please specify) _____________________________

6. Did you complete the accredited immunisation training at University?

Yes 🞏

No 🞏

If yes to question 6:

Please indicate your level of agreement with the statements below:

6a. The immunisation training was a valuable additional offering within the pharmacy degree

Strongly Agree 🞏

Agree 🞏

Neutral 🞏

Disagree 🞏

Strongly disagree 🞏

6b. The time commitment to complete the immunisation training at University was worthwhile

Strongly Agree 🞏

Agree 🞏

Neutral 🞏

Disagree 🞏

Strongly disagree 🞏

6c. The immunisation training offered at University influenced my decision to be actively involved in immunisations

Strongly Agree 🞏

Agree 🞏

Neutral 🞏

Disagree 🞏

Strongly disagree 🞏

6d. The immunisation training offered at University expanded opportunities for employment

Strongly Agree 🞏

Agree 🞏

Neutral 🞏

Disagree 🞏

Strongly disagree 🞏

6e. I believe other additional training/certifications would be useful during the pharmacy degree

Strongly Agree 🞏

Agree 🞏

Neutral 🞏

Disagree 🞏

Strongly disagree 🞏

If no to question 6,

6a Which of the following best describes your reasons not undertaking the immunisation training at University?

Unsure if it would interest me 🞏

Phobia of injections/needles 🞏

Intention to work overseas 🞏

Did not believe I would use this in my future work 🞏

Lack of time at University to complete 🞏

Other (please specify)________________________

6b Have you now completed accredited immunisation training? Yes 🞏 / No 🞏

If no – go to question 8

7. In regard to your current practice, which best describes the frequency of providing immunisations (on average):

Daily 🞏

Weekly 🞏

Monthly 🞏

Less than monthly 🞏

8. Have you become more actively involved in immunisations due to COVID-19?

Yes 🞏

No 🞏

Briefly describe your response:

______________________________________________________________________________________________________________________________________________________________________________________________________________________________

9. Do you currently have a role involving COVID-19 immunisations?

Yes 🞏

No 🞏

If yes to question 9:

9a Please describe your role (eg administration of vaccine, preparation of vaccine, etc):

________________________________________________________________________________________________________________________________________

If no to question 9:

9a Do you intend to become involved in COVID-19 immunisations?

Yes 🞏

No 🞏

Briefly describe your response:

____________________________________________________________________________________________________________________________________________________________________________________________________________

Please provide any additional comments you would like to make regarding the immunisation training. ________________________________________________________________________________________________________________________________________________________________________________________________________________________________________________________________________________________________________

***Thank you for your help with this research project.***
